# Connectivity analysis of ecological landscape networks by cut node ranking

**DOI:** 10.1007/s41109-018-0085-0

**Published:** 2018-07-31

**Authors:** Gianni Fenu, Pier Luigi Pau

**Affiliations:** Department of Mathematics and Computer Science, Via Ospedale 72, Cagliari, 09124 Italy

**Keywords:** Ecological networks, Network modeling, Connectivity

## Abstract

Ecological landscape networks represent the current paradigm for the protection of biodiversity. In the analysis of land features that precedes the establishment of land management plans, graph-theoretic approaches become increasingly popular due to their aptness for the representation of connectivity. Ecological corridors, seen as connecting elements for geographically distant areas dedicated to the preservation of endangered species, can be analyzed for the identification of critical land patches, by ranking cut nodes according to a score that encompasses various criteria for prioritized intervention.

## Introduction

Ecological landscape networks represent the current paradigm for nature protection policies throughout the world. The effectiveness of single natural reserves is enhanced by increasing the chances of migration of animals and dispersal of plants, thus merging the genetic pool of distant populations of endangered species, and improving biodiversity. In the European Union, the coordination of member states in the creation, maintenance and administration of a large-scale ecological network takes place within the Natura 2000 project: in accordance with European directives, municipalities and other local administrations are required to consider the impact of their land management decisions on conservation goals, at a national or continental scale. One of the relevant land management tasks in this context is that of proposing ecological corridors, aimed at improving connectivity between distant nature protection areas in order to enhance network behavior, particularly for plants and land animals.

The work of land managers is generally aided by Geographic Information System (GIS) tools, which are useful to store spatially referenced data and perform several kinds of analysis on the region of interest. In this and other contexts of operation, it is relevant to detect and analyze any network behavior in the set of land features. Thus, it is also common to build a graph representation of the features of interest, and apply complex network analysis techniques to complement the analysis performed with GIS tools. In the graph representation of a set of ecological corridors in an area of interest, cut nodes can be thought to correspond to critical patches.

The present work illustrates a method to rank cut nodes by performing connectivity analysis, which is meant to assist land managers in assessing priorities, with the identification of critical patches that require immediate attention. This article extends on previous work by the same authors (Fenu and Pau 2017), and introduces the definition of a combined score for cut nodes, which aims at providing land managers with a simple way to interpret results, compared to the previous method which required them to take into account multiple rankings. The exposition of the proposed method takes place with reference to a case study based on the area surrounding the Metropolitan City of Cagliari, located in southern Sardinia.

## Analysis of ecological networks

Land managers are tasked with pursuing diverse goals, often conflicting with one another, while considering a wide range of technical and political aspects. The goal of preserving or improving the state of green infrastructure may conflict with those concerning the maintenance of other existing infrastructure, such as the road network (Loro et al. 2015). Therefore, a mathematical model to represent ecological networks and enable quantitative analysis would be an invaluable help for land managers. In this section, the main concepts concerning ecological networks are defined, and a brief discussion of common analysis tools is given.

### Ecological networks

The traditional approach to the preservation of habitats and species is that of establishing natural reserves with specific conservation goals, but this has been deemed insufficient in many cases, particularly when reserves are too small or there is an excessive distance from other portions of habitat that are suitable for the protected species.

The current paradigm for nature protection is the creation of ecological networks (Jongman 1995). To this end, each nature protection area is to be designed to bring a valid contribution to large-scale preservation goals (Vimal et al. 2012). If it can be determined that the population of a species has the ability to migrate or disperse between reserves, meaning that separate populations can be considered to share their genetic pool, it is said that network behavior is present.

Migration between sites happens through so-called ‘ecological corridors’ (also referred to as ‘habitat corridors’ or ‘green corridors’). These can take different forms depending on which species is being considered: most land animals may require contiguous corridors, while flying birds may be able to migrate through paths made up of sets of disconnected patches (stepping stones), or even directly between sites when their distance is not excessive.

Network behavior may emerge from a favorable geographical configuration of natural reserves and habitat patches, or if necessary, be obtained by establishing and maintaining ecological corridors by human intervention.

### Analysis tools

Land features are commonly represented and analyzed using Geographic Information System (GIS) software tools, which allow an effective processing of spatial data and attribute data with geographical referencing. Two main forms of representation are possible for geographic features: as raster data, meaning that the region being analyzed is tessellated into a regular grid with a given cell size; or as vector data, meaning that lines and polygon boundaries are used to delimit areas of interest. The latter approach carries the advantages of better map precision, being able to work with boundaries of arbitrary shapes, and avoiding the problematic choice of a cell size; it remains true, however, that the most common kinds of analysis performed in this context perform better with raster data, and the storage of continuous attributes, such as altitude, is made easier by raster representations.

Graph models represent a complement to either approach, and are often employed to perform connectivity analysis. In the context of ecological networks, by connectivity it is usually meant that the migration or dispersal of a certain species is possible between areas, meaning that in a graph model, edges and their weights should be representative of the potential migrations of a chosen target species (or set thereof). Such a model is said to represent functional connectivity, and is generally preferred to models representing structural connectivity, based on inherent land properties (Urban et al. 2009).

In a context such as that of ecological landscape networks, preserving connectivity is often held as a priority. The identification of cut nodes or bridges, depending on the definitions given in creating a graph model and the features thereof, is the first step in determining which connecting elements require attention from land managers. The same principle has given rise to the application of graph-theoretic connectivity analysis in other contexts, including marine conservation (Treml et al. 2008), in which currents and other oceanographic features may act as ecological corridors, as well as different fields of application, such as wireless ad-hoc networks (Stojmenovic et al. 2011).

Concerning land management, depending on analysis goals and scale, each node in a graph model may represent an entire nature protection area (Mazaris et al. 2013), or a single habitat patch (Pereira et al. 2011). The two approaches are not necessarily mutually exclusive, and may complement one another, as the goal of a large-scale study can be that of understanding the global perspective of consequences of local decisions, or identifying an area that requires local intervention for the implementation of large-scale policies (Fenu et al. 2017). In performing graph analysis, it is important not to lose perspective, as the exclusive use of a graph-based approach may bring misleading results (Moilanen 2011); in order to avoid this, it is imperative to build a graph model in a way that accurately represents topological properties of the landscape.

In building a graph model, it seems natural to use edges to represent linking elements, such as ecological corridors, but in cases where the scale and the granularity of data allows for single land patches to be represented as nodes, it is sensible for the patches that make up ecological corridors to be also represented as nodes. Moreover, if a study is focused on birds, it is possible to link nodes representing areas within a given geographical distance, made to correspond to the dispersal distance of the species being examined. Generally speaking, edges are drawn to link pairs of nodes if migration has been detected or is considered possible between them. In cases where the possibility of migration and paths used by land animals are to be inferred, it is particularly useful to perform analysis based on the estimation of a resistance value on raster data representing land cells (McRae et al. 2008). Other techniques, which are also suitable for plant dispersal, are based on the comparison of genetic pools of distant population samples (Dyer and Nason 2004).

The applicability of complex network theory goes beyond connectivity analysis, as it is possible to calculate global and local network indices, in order to improve the understanding of properties of the ecological network (Bergsten and Zetterberg 2013). To mention a few examples, the clustering coefficient can be used to represent the degree of redundancy of links, and bottlenecks may be identified with various techniques, such as by ranking nodes according to their betweenness centrality index. This step is often performed as a part of vulnerability assessments, performed in various contexts of application, including ecological network themselves (Mazaris et al. 2013) as well as infrastructure networks, such as power grids (Fenu and Pau 2015).

## Case study

The method of analysis proposed in this article will be illustrated with reference to a case study, provided by a portion of an ecological network, located within the boundaries of the Metropolitan City of Cagliari. Information on the state of the network is obtained from data sets published within the Natura 2000 project managed by the European Union, and additional sources made available by the Region of Sardinia.

The European Union provides guidelines for the creation and maintenance of an ecological network across Europe within the scope of a project denominated “Natura 2000”. Nature protection areas can be recognized as Natura 2000 sites by going through a process in which they are designated as Sites of Community Interest (SCI), and later as Special Areas of Conservation (SAC). Areas of special interest for the protection of birds are designated as Special Protection Areas (SPA) and managed independently, and it is possible for a single site to hold the SPA designation as well as the SCI or SAC one, and for an area with SPA designation to overlap a site recognized with SAC or SCI status. However, there is no specific regulation for the designation of ecological corridors. For this reason, it was necessary to complement Natura 2000 data with additional sources.

The following data sets were used:

- public databases made available within the Natura 2000 project, with information on recognized Natura 2000 sites, and a list of species of interest for the project;
- a land cover map provided by the Region of Sardinia in year 2008, based on CORINE land use codes, which make it possible to identify agricultural and urban areas, and distinguish between different kinds of vegetation in natural and semi-natural areas;
- a set of habitat suitability values referred to each land use code for each species of interest, also made available by the Region of Sardinia.

The identification of ecological corridors was performed by assessing the suitability as green infrastructure of land patches, and their contribution to habitat fragmentation (Cannas and Zoppi 2017). The aggregation of suitability values and other ecological values is the basis for the creation of a resistance map, where land cells are associated with a ‘cost-weighted distance’ (CWD) value; for this study, land patches within the first decile of CWD values are selected as suitable patches for ecological corridors. Least-cost paths for the migration of species can also be identified on the map; the combination of patch selection and identification of paths between Natura 2000 sites is used to identify potential habitat corridors in the area.

The corridors thus identified represent a scope of analysis for land managers; possible activities include an assessment of their condition with a local evaluation, and a verification of whether any migration actually takes place on them. In that case, it should be determined whether any threat to their continued existence can be detected; otherwise, reasons for the lack of migration should be inferred, and it can be sensible to determine whether it is possible to take action to enable corridors within the identified land patches.

**Fig. 1:**
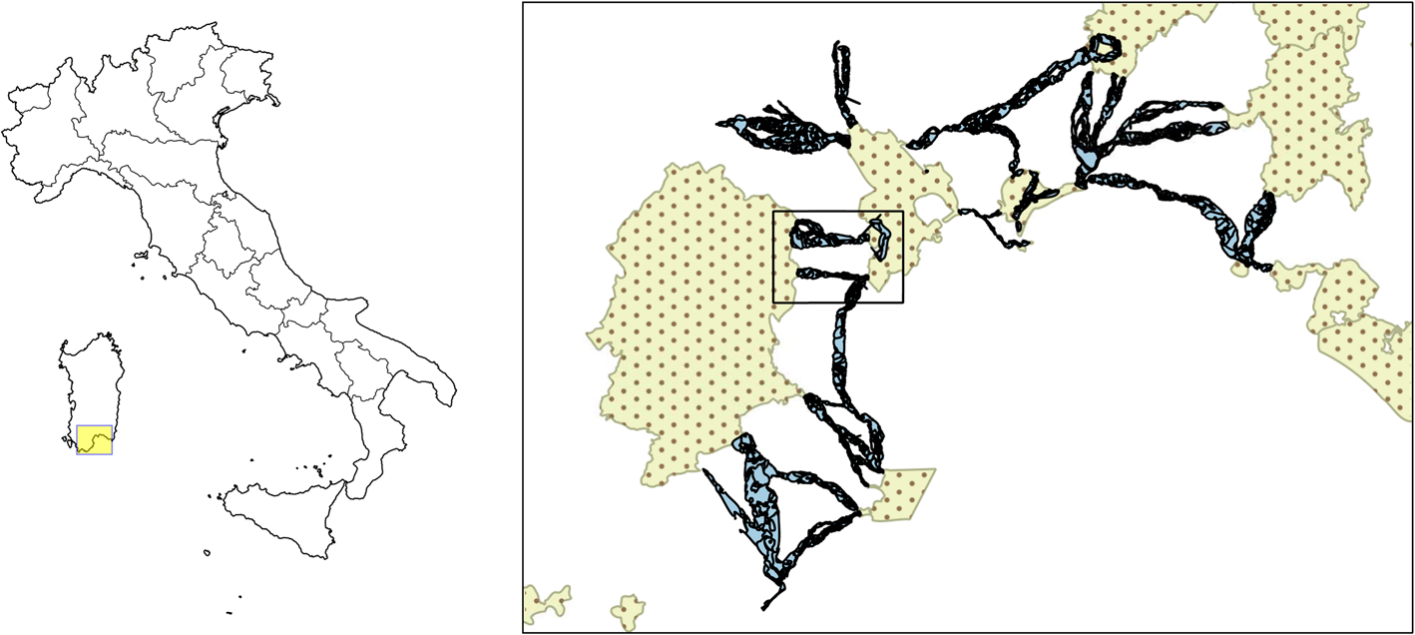
Left: a map of Italy. The highlighted box marks the area represented on the right. Right: map of Natura 2000 sites (dotted areas) and proposed corridors in the Metropolitan City of Cagliari, Sardinia. The analysis was performed for the entirety of the corridors represented here. The superimposed box corresponds to the smaller area shown in Fig. 4 and others

The analysis presented in this article was performed for the whole area of the Metropolitan City of Cagliari. The results that will be discussed concern the entirety of this area; some geographically referenced results were shown for a portion of this area (see Fig. 1). These are meant to provide an example of what kind of map can be produced for the entire area as an aid to land managers.

## Graph model building and analysis

A necessary preliminary step for the connectivity analysis is to build a graph model of the proposed ecological corridors. First, the land cells that make up these corridors, identified as described in the previous section, are intersected with the original patches from the land cover map, producing a vector map, in which land patches are homogeneous from the point of view of land use codes. The node set shall correspond to this set of land patches. A spatial graph model is built by linking pairs of nodes when they correspond to pairs of adjacent patches on the map.

For this case study, the graph model built with this process has 6995 nodes and 17464 edges, organized in 18 connected components (the number of nodes in each component is reported in Table 1). Each component corresponds either to a single candidate corridor between Natura 2000 sites, or to a connected set of candidate corridors, which form multiple bridges to connect sites.

**Table 1:** Number of nodes in each original connected component

Component	Number of nodes
1	2464
2	1177
3	975
4	796
5	681
6	298
7	224
8	159
9	62
10	57
11	47
12	19
13	12
14	7
15	6
16	5
17	3
18	3

### Method of analysis

The identification of cut nodes, i.e. those nodes that determine the separation of a connected component into two or more smaller components when removed, can be considered a first step in assessing the robustness of a corridor set and ranking critical patches, which may require further inspection.

While this seems straightforward enough, the identification of cut nodes turns out to be of little use by itself, as the method to build a graph model for a set of ecological corridors is such that cut nodes can be expected to be abundant by construction. In this study, out of 6995 nodes in the graph model, 569 are cut nodes (about 8.13%); clearly, it is not very helpful to have this number of cut nodes reported as corresponding to critical habitat patches. It can also be observed that the problem is only made worse by the fact that the land cover map has a large number of enclaves, i.e. patches that are topologically surrounded by another patch. Enclaves translate to leaf nodes in the graph model, so each patch surrounding one is counted as a cut node, even in situations where its removal does not cause an interruption of an ecological corridor in the underlying map. These cut nodes can be interpreted as noise, especially when the leaf node corresponds to a very small patch, which may be due to artifacts in the source spatial data.

These observations suggest that the identification of cut nodes has to be followed by the application of some criterion to rank cut nodes by priority, or to filter out those of little interest for land managers. For this purpose, the removal of each cut node is simulated, in order to record relevant measures corresponding to each node removal:

- the number of additional connected components that result from removal of the node;
- the size of each new connected component.

Formally, let *G*^′^=(*V*^′^,*E*^′^) be a connected component of graph *G*, and let *v*∈*V*^′^ be a cut node in the component *G*^′^. The removal of *v* results in the split of *G*^′^ into *n* smaller components $G^{\prime }_{k} = \left (V'_{k}, E'_{k}\right)$ with *k*∈{0,1,...,*n*−1}, and *n*≥2. Choose indices such that |*V*0′|≥|*V*1′|≥...|*Vn*−1′|.

Define the *additional component count* of a cut node as

1 $$ C_{A}(v) = n-1,  $$

corresponding to the number of components that are added to the total for the graph when the cut node is removed; and the *second-size* of a cut node as the size of the second-largest component it generates, that is,

2 $$ C_{S}(v) = \left|V'_{1}\right|.  $$

This definition is such that the second-size attribute is 1 for a cut node if its removal results in the disconnection of one or more leaf nodes from a single larger component; in this context, this is useful to identify cut nodes that are most likely to be noise, under the assumption that a disconnected leaf node may be an enclave due to artifacts in the original data.

Other measures associated to cut nodes can be considered. In this study, a normalized betweenness centrality index of each node will be calculated for inclusion in the definition of a combined score. Recall that the betweenness centrality index of a node *v* is defined as:

3 $$ C_{B}(v) = \sum_{s \neq v \neq t \in V} \frac{\sigma_{st}(v)}{\sigma_{st}},  $$

where *σ*_*st*_(*v*) is the number of shortest paths from *s* to *t* that include *v*, and *σ*_*st*_ is the total number of shortest paths from *s* to *t*. This measure can be normalized to a scale from 0 to 1 by dividing the number of node pairs excluding *v*. For simple unweighted graphs, this is given by

4 $$ \frac{(N-1)(N-2)}{2},  $$

where *N*=|*V*|. The normalized version of the betweenness centrality will be used in this study.

Lastly, it may also be of interest to make use of additional measures, not based on graph properties. One such example is the minimum geographical distance from a Natura 2000 site. Formally, if *S* is the set of Natura 2000 sites, this measure is given by

5 $$ D(v) = min_{s \in S} \, d(s, v),  $$

where *d* expresses the geographical distance, calculated between boundaries of land patches.

The entire process of ranking nodes can be summarized as follows:

1. build a graph model, by instantiating a node for each homogeneous patch, and connecting nodes corresponding to adjacent patches on the map;
2. identify the set of connected components in the graph model, treating each component separately in the remainder of the process;
3. for each component, identify its set of cut nodes, and note its geographical distance from a site;
4. perform the removal of each single cut node, noting the number of additional components resulting from the split (*C*_*A*_) and their second-size (*C*_*S*_) as attributes of the cut node;
5. rank patches according to one of their attributes, or a combination of these attributes, complex network indices (such as the betweenness centrality), and other measures (such as the distance of the patch from a nature preservation site).

For this study, steps 1 and 5 were performed with custom Python scripts developed for use within the QGIS software suite (QGIS Development Team 2009); code for the remaining steps was implemented in Java, using the open source library JGraphT for graph representation and analysis^1^. The connectivity inspector included within JGraphT was used for the identification of connected components (steps 2 and 4), while the biconnectivity inspector was used to identify cut nodes (step 3). QGIS was also used to export maps as images. Calculations of the betweenness centrality index were performed with Cytoscape version 3.6.0 (Shannon et al. 2003). Geographical distances (as part of step 3) were calculated by importing data into a SQLite database and using the Spatialite extension for spatial analysis. All distances are calculated on map projections, and are to be treated as an approximation for this reason; however, at this scale, the degree of distortion can be considered small enough to be negligible.

### Choice of a combined score

A discussion of the distribution of the *additional component count* and *second-size* attributes will be helpful in supporting the choice of a combined score for the ranking of patches.

**Fig. 2:**
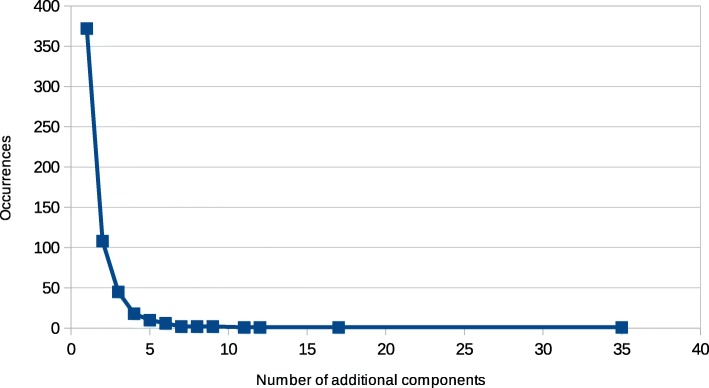
Statistical distribution of the additional component count resulting from the removal of each cut node

Table 2 reports the occurrences of additional component counts for all cut nodes in the considered area in the Metropolitan City of Cagliari. A majority of cut nodes (372, i.e. over 65% of the total number of cut nodes) split their corresponding component into two new ones (i.e. 1 additional component), while 108 cut nodes split their component into three parts (2 additional components). After these, the number of occurrences of higher figures quickly drops; the statistical distribution is visualized in Fig. 2. The percentile score reported in the table is discussed later.

**Table 2:** Occurrences of additional component count resulting from the removal of cut nodes

Additional components	Occurrences	Percentile
1	372	0.00000
2	108	65.37786
3	45	84.35852
4	18	92.26714
5	10	95.43058
6	6	97.18805
7	2	98.24253
8	2	98.59402
9	2	98.94551
11	1	99.29701
12	1	99.47276
17	1	99.64851
35	1	99.82425

**Fig. 3:**
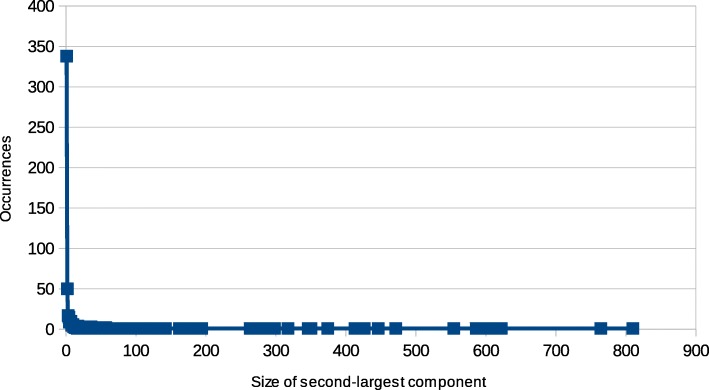
Statistical distribution of size of second-largest component resulting from the removal of each cut node

The second-size attribute behaves similarly. There are 338 cut nodes (over 59%) which only disconnect leaf nodes if removed, 50 that disconnect pairs of nodes, etc. (see Table 3), with an even steeper drop for the number of occurrences of sizes larger than 1. The largest second-size value with at least two occurrences is 57, and the global maximum is 810. The complete distribution is visualized in Fig. 3.

**Table 3:** Occurrences of size of second-largest component resulting from the removal of cut nodes (excerpt)

Size of component	Occurrences	Percentile
1	338	0.00000
2	50	59.40246
3	17	68.18981
4	15	71.17750
5	9	73.81371
6	10	75.39543
7	10	77.15290
8	5	78.91037
9	5	79.78910
10	6	80.66784
11	3	81.72232
12	3	82.24956
...	...	...
764	1	99.64851
810	1	99.82425

An attempt to summarize the considered attributes into a single value could be made by computing a summation or a linear combination. However, as the measures are not in the same scale of values, a problem lies in the fact that one of the measures could dominate the other ones. To address this issue with finding a meaningful aggregated score value to rank cut nodes and assess priorities, it is possible to make use of a combination of percentiles, so that each measure is brought to a comparable scale from 0 to 100.

Multiple definitions and interpretations of percentile scores exist. Since the goal is to filter out patches with the smallest possible values for the component count and component size measures, the interpretation that was selected for this study is the percentage of values that are strictly less than the measure^2^. This excludes 100 as a possible value, while assigning a score of 0 for patches with the smallest value (see Tables 2 and 3), avoiding a contribution to the combined score, which is a desirable behavior in this context. At the same time, there is no significant difference in the percentile score of the top values, regardless of the actual difference in the raw values, and this is also consistent with the goal of identifying patches with abnormally high attribute values, without giving too much importance to the actual value.

### Results

**Fig. 4:**
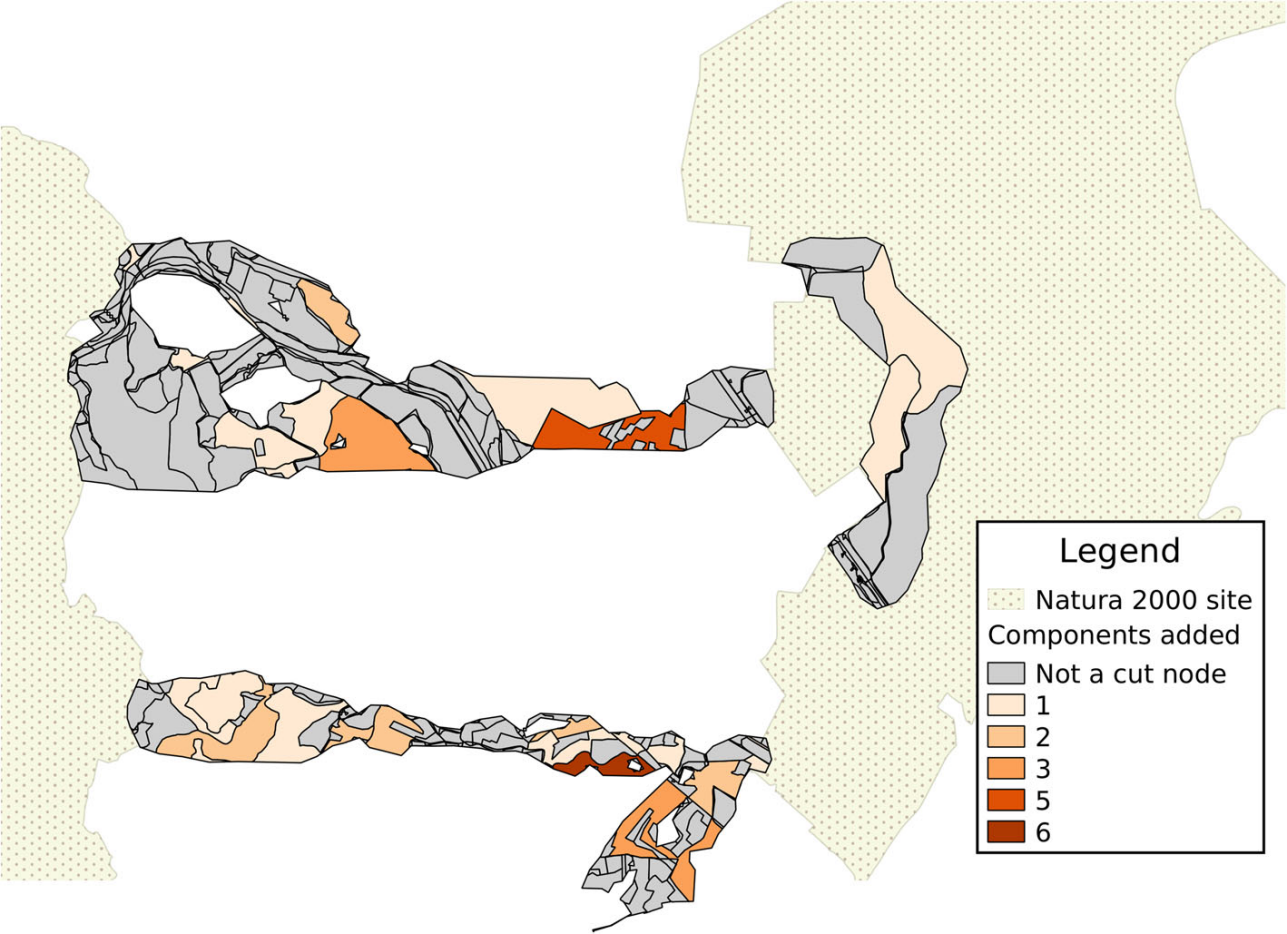
A darker shade represents a higher count of components resulting from the removal of a cut node

**Fig. 5:**
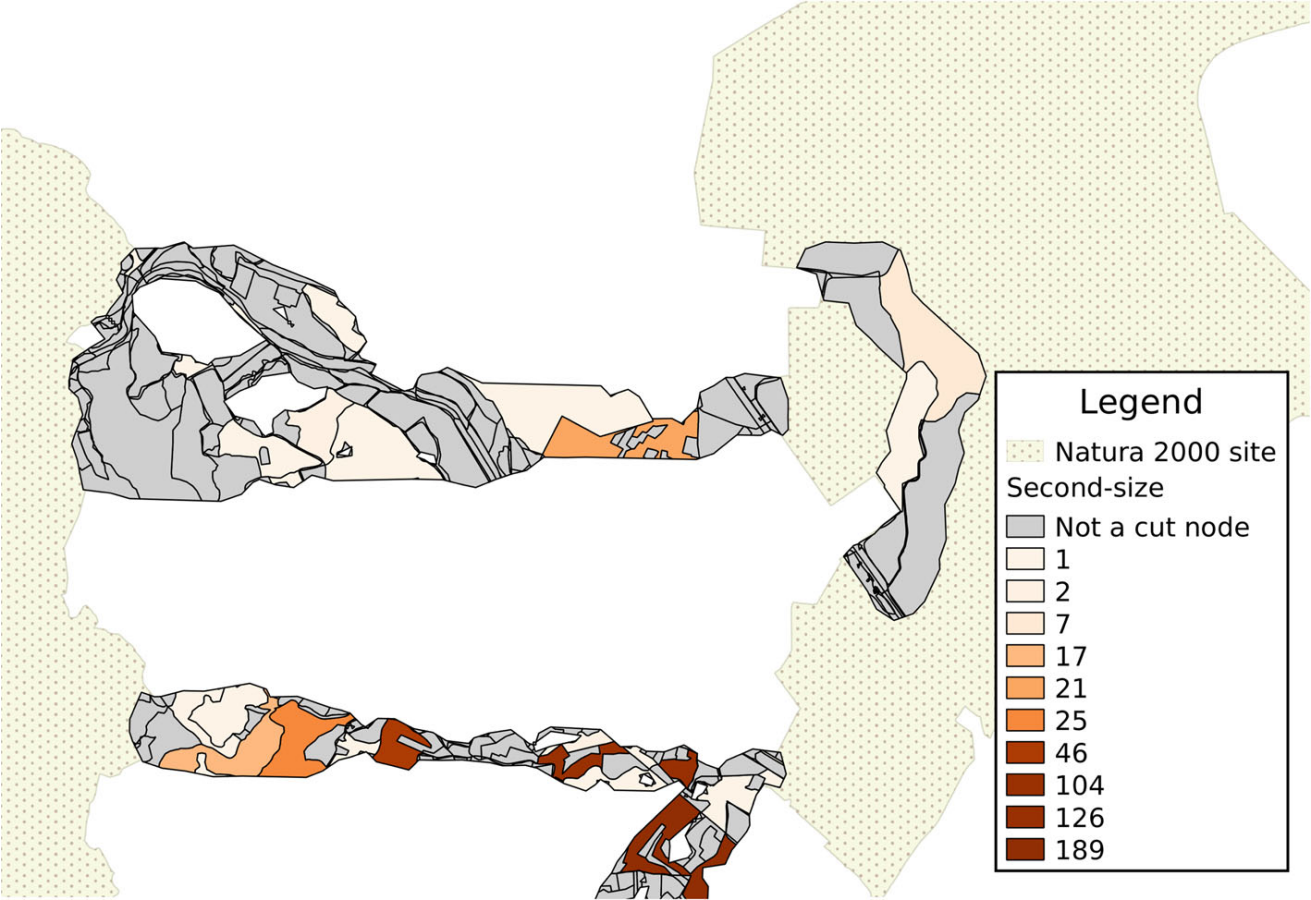
Cut nodes are ranked according to the size of the second-largest component resulting from their removal. A darker shade represents a larger size

**Fig. 6:**
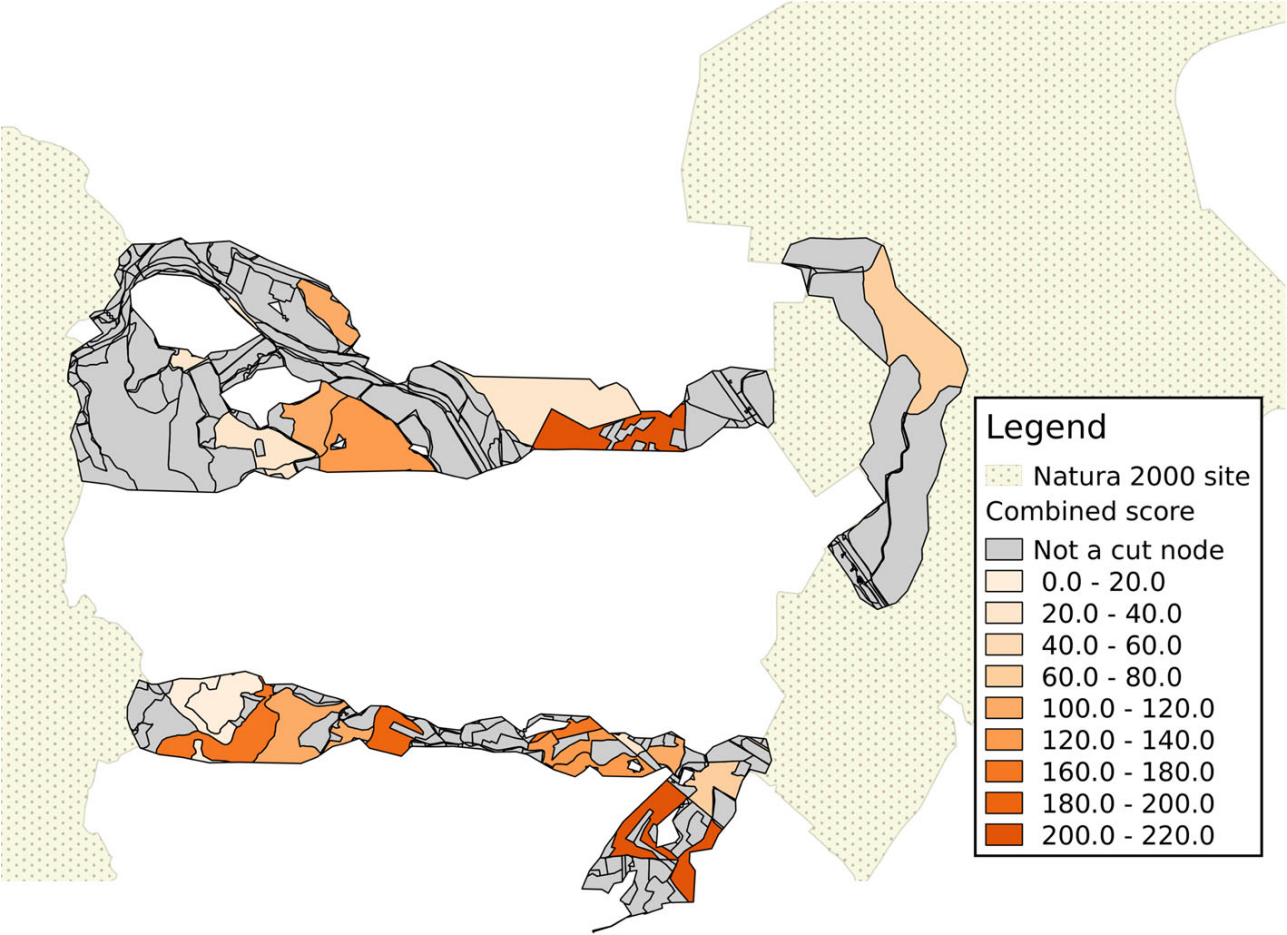
Combined score for habitat patches. The score corresponds to the sum of percentiles of three values: component count, second-size and minimum distance from a Natura 2000 site

An example of visualization of results is provided for a portion of the area being analyzed in Figs. 4, 5 and 6. In each map, the shades of color are darker for patches corresponding to cut nodes with higher scores or attribute values, with patches marked in grey not corresponding to a cut node. In the remainder of this section, the component lying closest to the North-West corner of the represented area is used as an example of how results can be interpreted. This is the sixth-largest component in the study, with 298 nodes, and 13 cut nodes.

Table 4 lists the cut nodes detected in this component. For each node, identified by a number assigned progressively to every node, the table reports the three relevant measures (number of components created with its removal, size of second-largest component, and minimum distance from Natura 2000 sites), together with their percentile score according to the definition provided earlier. The combined score, corresponding to the sum of the three percentile scores, is also reported. In the last two columns, the normalized betweenness centrality index of each node is reported, together with the rank order of cut nodes by betweenness, for comparison (the table is ordered by descending combined score).

**Table 4:** Excerpt of indicators of detected cut nodes in a connected component. The value and percentile are provided for component count, second-size and distance from a site. The aggregated score is the sum of percentile scores

Node	Comp. count		Second-Size		Distance		Combined	Betweenness C.	
ID	Value	Pctl.	Value	Pctl.	Value	Pctl.	Score	Value	Rank
1949	5	95.43	21	85.06	532.0	21.62	202.11	0.17130	2
6884	3	84.36	1	0.0	1997.8	45.17	129.53	0.06987	5
4383	2	65.38	1	0.0	1469.3	37.79	103.16	0.01674	9
6869	1	0.0	2	59.40	1684.5	41.48	100.88	0.05562	6
6889	1	0.0	1	0.0	2855.0	55.89	55.89	0.00980	11
6885	1	0.0	1	0.0	1806.8	43.41	43.41	0.00673	13
5541	1	0.0	1	0.0	1489.6	38.31	38.31	0.01513	10
5094	1	0.0	1	0.0	1039.6	30.40	30.40	0.07742	4
2712	1	0.0	1	0.0	952.3	28.30	28.30	0.19063	1
1607	1	0.0	1	0.0	860.3	26.36	26.36	0.03149	8
2638	1	0.0	1	0.0	681.1	23.90	23.90	0.08808	3
1604	1	0.0	1	0.0	0.0	0.0	0.00	0.00675	12
4579	1	0.0	1	0.0	0.0	0.0	0.00	0.04956	7

The cut node with the highest combined score is the one with ID 1949, which outranks the other nodes both for the component count attribute and the second-size one. In this section of the map, the top three cut nodes by combined score are the same nodes that occupy the first three positions if nodes are ranked by the additional component count, but this behavior is not observed in the general case, and the use of a combined score is still justified for the end result as it allows a definite priority list to be presented to land managers.

Results can be visualized on a map, by using a color gradient to represent either the component count (Fig. 4) or the second-size (Fig. 5); with a proper choice of scale, it is easier to identify land patches with a higher score (the patch with the the darkest shade of color in the north-western component, in both figures, corresponds to the node with ID 1949). Once again, the combined score makes it possible to aggregate the visualization into a single map (Fig. 6).

**Fig. 7:**
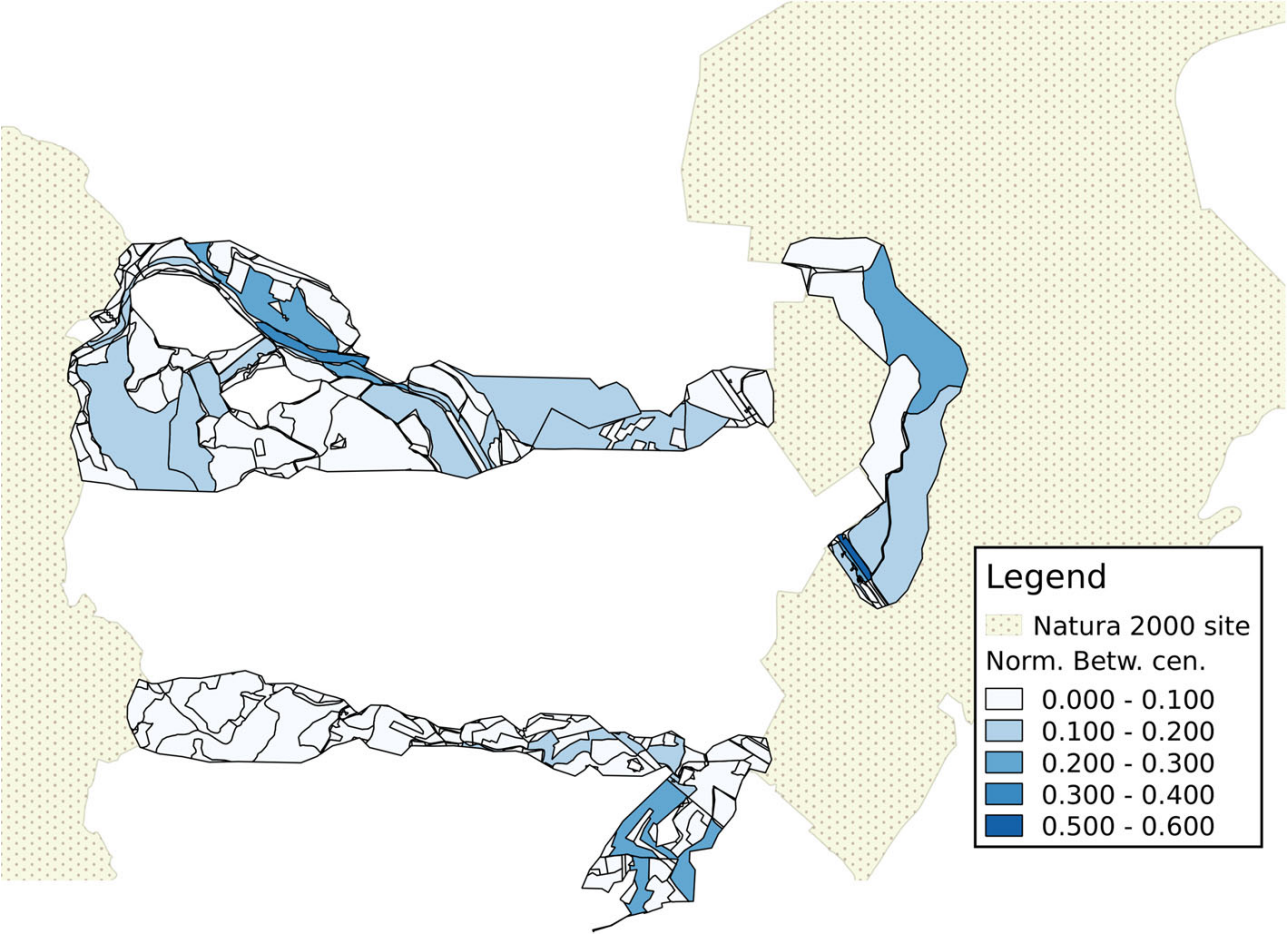
Cut nodes are ranked according to node betweenness centrality. A darker shade represents a larger value

**Fig. 8:**
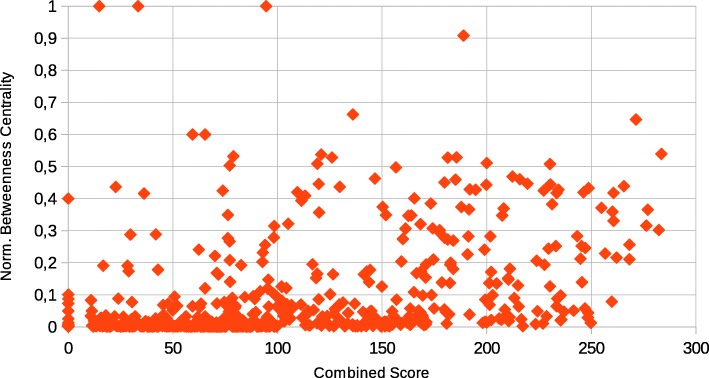
Plot of the correlation of combined score (X-axis) and betweenness centrality (Y-axis)

A comparison with the betweenness centrality (Fig. 7) shows that the combined score defined in this work behaves differently from this index. To support this notion, a plot of the values is shown in Fig. 8, with the combined score in the X-axis and the normalized betweenness centrality in the Y-axis. The Pearson correlation index of the two variables is 0.37585, and the Spearman correlation index is 0.50343; these values do not suggest that a strong correlation exists between the two variables.

## Conclusions and future work

Current policies for the preservation of nature and biodiversity are based on the concept of ecological landscape networks, made up of ecological corridors and nature protection areas. In situations where conservation goals include the establishment or maintenance of contiguous ecological corridors, the identification of critical land patches and use types may have an influence on decisions concerning land management plans, which are required to take into account the consequences on nature conservation goals, while implementing infrastructural changes.

Ecological corridors are not thoroughly regulated within the Natura 2000 project. Because of this, they have to be identified or proposed by local administration, and external data sources are required to represent them in a form that may be analyzed. In this article, a case study based on data pertaining to the Metropolitan City of Cagliari was discussed, in order to show how a graph model can be created from land use data, representing homogeneous land patches with nodes. With a subsequent identification of cut nodes, critical patches and land use types can be determined; however, further steps are necessary to rank cut nodes and assess priorities, because of their number. The criteria to rank cut nodes are not easily defined, as several measures can be defined and it is not straightforward to combine them into a single ranking value. A simple proposal was presented and discussed in this article; while the method has a potential for being used in applications, the refinement of ranking values remains a goal for future research. Further developments may also include experimenting with the simultaneous removal of several cut nodes.

## Abbreviations

*C*_*A*_(*v*): Additional component count of a cut node *v*
*C*_*B*_(*v*): Betweenness centrality index of a node *v*
*C*_*S*_(*v*): Second-size of a cut node *v* (size of second-largest component obtained by its removal)
*D*(*v*): Minimum geographical distance of a node *v* from a site
CWD: Cost-weighted distance
GIS: Geographic information system
SAC: Special area of conservation
SCI: Site of community interest
SPA: Special protection area

## Footnotes

1 JGraphT is available from http://jgrapht.org/

2 In Python, this is implemented as the ‘strict’ interpretation of percentiles in the scipy.stats.percentileofscore method.
